# Knowledge of hepatitis B infection, hepatitis B vaccine, and vaccination status with its associated factors among healthcare workers in Kampot and Kep Provinces, Cambodia

**DOI:** 10.1186/s12879-024-09571-y

**Published:** 2024-07-01

**Authors:** Savoeun Sok, Chanroth Chhoung, Bunlorn Sun, Ko Ko, Aya Sugiyama, Tomoyuki Akita, Shingo Fukuma, Junko Tanaka

**Affiliations:** 1 Department of Epidemiology, Infectious Disease Control and Prevention, Graduate School of Biomedical and Health Sciences, Hiroshima University, 1-2-3, Kasumi, Minami-Ku, Hiroshima, 734-8551 Japan; 2 Project Research Center for Epidemiology and Prevention of Viral Hepatitis and Hepatocellular Carcinoma, Hiroshima University, Hiroshima, Japan; 3 Kep Provincial Health Department, Kep, Cambodia; 4 Kampong Speu Provincial Health Department, Kampong Speu, Cambodia

**Keywords:** Hepatitis B, Vaccination status, Healthcare workers, Kampot, Kep, Cambodia

## Abstract

**Background:**

Healthcare Workers (HCWs) are susceptible to hepatitis B virus (HBV) infection and are advised to receive vaccination. However, vaccination rates remain low in developing countries. There is little data concerning Hepatitis B (HepB) vaccination and information regarding HBV knowledge among HCWs in Cambodia. This study aimed to evaluate the knowledge of HBV infection, HepB vaccine, and vaccination status with its associated factors among HCWs in Cambodia.

**Methods:**

A Cross-sectional study was conducted among HCWs in Kampot and Kep Provinces, Cambodia, from September to October 2023 using a questionnaire survey. A total of 261 HCWs were recruited from 1,309 individuals working in all 83 health facilities using systematic random sampling methods. Statistical analyses including the χ^2^-test and multivariate logistic regression were conducted to identify factors associated with vaccination among the participants.

**Results:**

Among 259 participants, 62.9% showed good knowledge of HBV infection, and 65.6% demonstrated good knowledge of the HepB vaccine. 59.8% of the participants had received the HepB vaccine, while 40.2% remained unvaccinated. Analysis showed that HCWs working at Provincial Health Department/Operational Districts and Provincial Referral Hospital/Referral Hospitals were more likely to be vaccinated compared to those at Health Centers [AOR = 6.5; CI = 1.1–39.5,* p* = 0.0403; AOR = 2.8, CI = 1.0–7.8, *p* = 0.0412], respectively. Furthermore, individuals with good knowledge of the HBV infection and vaccine were more likely to receive the vaccine compared to those with inadequate knowledge [AOR = 6.3; CI = 3.3–12.3,* p* < .0001; AOR = 3.7, CI = 1.9–7.4, *p* = 0.0001], respectively. Within the unvaccinated HCWs, 32% reported high vaccine costs as a barrier, 33% mentioned workplace vaccine was not for adults, and 59% reported insufficient education on adult HepB vaccination.

**Conclusions:**

The HepB vaccination coverage among HCWs is at 59.8%, which is below the World Health Organization’s (WHO) recommendation rate of 100%. Knowledge of HBV infection and HepB vaccine were good predictive factors for vaccination. The high cost of vaccine, workplace vaccine not for adults, and insufficient education on adult vaccination were found as barriers to vaccination. This study underscores the importance of providing education to HCWs on HBV infection and the HepB vaccine. Furthermore, it highlights the need for a policy that ensures free vaccination for HCWs.

**Supplementary Information:**

The online version contains supplementary material available at 10.1186/s12879-024-09571-y.

## Background

Hepatitis B virus (HBV) infection is a major problem threatening​ health globally. Individuals with the infection are at risk of developing chronic hepatitis B (CHB), which can lead to liver cirrhosis and hepatocellular carcinoma (HCC) with a high risk of mortality [1]. In 2019, there were 1.5 million new infections annually, with 296 million people carrying chronic infections and 820,000 deaths reported worldwide. The highest prevalence of HBV is observed in the World Health Organization (WHO) region of the Western Pacific, where there are 116 million infections [1]. A nationwide study conducted in Cambodia in 2017 revealed that HBV prevalence was 0.56% in children aged 5–7 years and 4.39% in their mothers [2]. Cambodia achieved the WHO regional goal by reducing the prevalence of HBV infection in 5-year-old children to under 1% by 2017 through the implementation of a nationwide HepB vaccination program [3, 4].

Healthcare Workers (HCWs) are at risk of acquiring HBV infection due to accidental exposure to infected blood and body fluids [5–9]. Globally, 3 million HCWs experience exposure to blood pathogens yearly, 2 million of those exposed to HBV. Surprisingly, over 90% of these incidents happen in developing countries [10]. Annually, there are 66,000 cases of HBV infection among HCWs, resulting in 261 deaths globally. Notably, 40% of HBV infections among HCWs are attributed to accidental occupational exposure [11].

Several studies have demonstrated a concern on lack of knowledge regarding HBV infection and the HepB vaccine among HCWs in developing countries. A study carried out in Ethiopia in 2012 found that only 52% of HCWs had adequate knowledge of HBV infection, and 62% had good knowledge of the HepB vaccine [6]. In Nigeria, a 2017 study revealed that 82.9% of HCWs had a good understanding of HBV infection, while only 44.5% were knowledgeable about the HepB vaccine [12]. Additionally, a nationwide study in Cambodia in 2019 found that only 17.4% of HCWs were aware that HBV can be transmitted from mother to child during birth, and only 37.1% knew that a full dose of the HepB vaccine can protect for at least 20 years [13].

The HepB vaccine is known to be safe and effective in preventing HBV infection and is recommended for all HCWs. Receiving a full dose of the vaccine can provide individuals with nearly 100% protection against HBV infection and its consequences [1]. All countries in the region of the Western Pacific were expected to develop a national policy of HepB vaccination for HCWs by 2020 [14]. However, to date, Cambodia has not established such a policy. There is little data on HepB vaccination among HCWs in Cambodia. In addition, current information regarding the knowledge of HBV infection and the HepB vaccine among them remains limited. Understanding their knowledge will inform targeted educational interventions, while comprehensive vaccination information is crucial for informing policymakers. Therefore, this study was conducted to evaluate the knowledge of HBV infection, HepB vaccine, and vaccination status with its associated factors among HCWs.

## Methods

### Study setting and population

The healthcare system in Cambodia is divided into 3 levels: National, Provincial, and Operational District levels. The Provincial and Operational District levels are present in each province or municipality. The Provincial level comprises Provincial Health Departments (PHDs) and Provincial Referral Hospitals (PRHs), while the Operational District level encompasses Operational Districts (ODs), district-based Referral Hospitals (RHs), Health Centers (HCs), and Health Posts (HPs). PHDs and ODs primarily handle healthcare administration while PRHs, RHs, HCs, and HPs are responsible for providing healthcare services [15].

A cross-sectional study was conducted from September 1, 2023, to October 31, 2023, among HCWs in the public healthcare sector of Kampot and Kep Provinces, Cambodia. These coastal provinces, situated in the southwestern of the country, are popular tourist destinations for both national and international visitors [16, 17]. Additionally, their proximity to Vietnam results in significant population movement, potentially increasing the risk of HBV transmission and posing a threat to HCWs. Thus, these provinces were purposefully selected from the twenty-five provinces and municipalities. These two provinces covered a total of 83 health facilities (HFs). Kampot has 76 facilities, comprising one PHD, one PRH, four ODs, four RHs, and sixty-four HCs. Kep, on the other hand, has 7 HFs, consisting of one PHD, one PRH, and five HCs [18]. According to administrative offices, there are 1,309 HCWs in total, with 1,173 in Kampot Province and 136 in Kep Province. The HCWs included in the study consist of physicians, nurses, midwives, laboratory technicians, pharmacists, dentists, radiologists, and administrative staff.

### Sample size and calculation

The required sample size for this study was calculated using JMP® Pro V.17.0.0 (SAS Institute, Cary, NC, USA), with the following assumptions: an expected vaccination coverage of 83% among HCWs in Cambodia, based on a study conducted in a neighboring country (Vietnam) [19], a 95% confidence interval, a margin of error of 5%. Assuming a non-response rate of 20%. The estimated sample size of HCWs needed for the study is 261.

### Sample recruitment

**Fig. 1:**
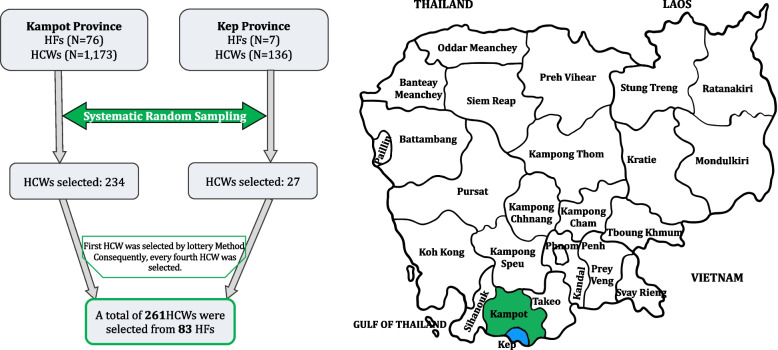
Flowchart of sampling procedure**.** The flowchart illustrates the recruitment of subjects from all health facilities (HFs) in Kampot and Kep Provinces between September and October 2023. Of the 83 HFs, 76 are in Kampot Province, and 7 are in Kep Province. A total of 1,309 healthcare workers (HCWs) were identified, with 1,173 from Kampot and 136 from Kep Province. From these, 261 HCWs were randomly selected

A systematic random sampling method was used to select 261 HCWs out of 1,309 HCWs from 83 HFs listed by the administrative offices of Kampot and Kep Province. These HCWs were working in offices, wards, and rooms of each HF. We initiated the systematic random sampling by dividing the total of 1,309 HCWs (N) by the desired sample of 261 (n), resulting in a sampling interval of five (k). The first participant was randomly selected using a lottery method and it was the fourth HCW. Thereafter, every fifth HCW was selected, starting from the fourth, until a total of 261 participants was reached [20] as shown in (Fig. 1). Eligible HCWs who were actively engaged in professional duties or community fieldwork were invited to participate at their convenience. In cases where an HCW declined to take part, the next individual on the list was invited, typically the one following the individual who refused. Those who were on maternity leave or those pursuing education abroad were excluded from the study.

### Data collection

The questionnaires were adapted from a previous study [6] and comprised four parts: socio-demographic characteristics, knowledge of HBV infection, knowledge of the HepB vaccine, and vaccination status. Initially composed in English, the questionnaires were later translated into Khmer by native Khmer speakers who also speak English. A pre-test involving ten HCWs in Kep was conducted to ensure the questionnaires were clear and understandable. Each participant was asked ten questions on knowledge of HBV infection and fourteen questions on knowledge of the HepB vaccine, scoring 1 point for a correct response and 0 for an incorrect answer or if they did not know.

The level of knowledge about HBV infection and the HepB vaccine was assessed as either "good" or "inadequate" based on the mean score. Participants scoring above the mean were categorized as having good knowledge, while those scoring below the mean were categorized as having inadequate knowledge [6]. Additionally, HepB vaccination status was self-reported, and individuals who received three or more vaccine doses were considered fully vaccinated [21].

The study's objectives, data collection methods, and potential benefits were presented to the Directors of PHDs. Upon obtaining their approval, we enlisted the help of chief administrative officers to distribute official letters to all HFs under their supervision via a designated Telegram group. Subsequently, we visited each HF and briefed the facility chiefs about the study. HCWs selected from the roster were given detailed information about the study and invited to participate. After the consent was obtained from each voluntary participant, paper-based self-administered questionnaires were distributed. All respondents were closely monitored to ensure they did not reference external sources during the 20-min questionnaire completion period. Upon collecting the questionnaires, we checked for completeness and ensured that no question was left unanswered. The collected data were then entered into a Microsoft Excel spreadsheet, with a double-check to minimize the chances of data missing.

### Data analysis

JMP® Pro V.17.0.0 (SAS Institute, Cary, NC, USA) was employed for both data cleaning and analysis. Categorical variables were presented as frequencies and percentages, while continuous variables were described using the mean and standard deviation (SD). χ^2^–test and multivariable logistic regression analyses were performed to identify factors associated with knowledge of HBV infection, knowledge of the HepB vaccine, and vaccination status among participants. Based on previous research that highlighted age as a significant factor related to the knowledge of HBV infection and vaccination status among HCWs [19, 21–25], age was assumed as a potential confounding variable and constantly included in the model. The remaining eight variables were chosen for inclusion in the multivariable model using the stepwise method. Adjusted odds ratios (AOR) and their 95% confidence intervals (CI) were calculated to quantify the strength of the association. A p-value < 0.05 was considered statistically significant.

## Results

### Characteristics of participants

Out of the 261 healthcare workers (HCWs) invited, 259 from 83 HFs agreed to participate in the study, resulting in a response rate of 99%. Among the participants, 89.6% were from Kampot and 10.4% from Kep Province. The mean age of the participants was 37.6 years, with a standard deviation (SD) of 8.9 and a range of 24 to 59 years. Females accounted for 55.6% of the participants. Nurses and midwives made up the largest proportion of participants, at 40.1% and 38.2% respectively. All participants were employed at PHD/ODs, PRH/RHs, and HCs, constituting 18.5%, 30.9%, and 50.6%, respectively. Approximately 32% of them worked in obstetric or delivery units, while 38% were assigned to the Outpatient Department (OPD) or other units. The mean year of work experience was 13.3 years, with a standard deviation of 9.5 and a range of 1 to 40 years (Table 1).

**Table 1:** Socio–demographic characteristics of healthcare workers in Kampot and Kep, Cambodia

Characteristics	Frequency N = 259	Percentage %
**Age:** mean age is 37.6 years (SD: 8.9; range: 24–59 years)		
≤ 30	57	22.0
31–40	132	51.0
41–50	33	12.7
> 50	37	14.3
**Gender**		
Male	115	44.4
Female	144	55.6
**Occupation**		
Physician (MD/MA)	37	14.3
Nurse	104	40.1
Lab technician	9	3.5
Midwife	99	38.2
Others (Pharmacist & Administrator)	10	3.9
**Province**		
Kampot	232	89.6
Kep	27	10.4
**Workplace**		
PHD/ODs	48	18.5
PRH/RHs	80	30.9
HCs	131	50.6
**Working unit**		
Technical office	28	10.8
Admin/account office	11	4.2
Emergency/ICU/Surgery/Lab/Pediatric	40	15.4
Obstetric/Delivery unit	82	31.7
OPD/Other	98	37.8
**Experience:** mean years of practice is 13.3 years (SD: 9.5, rang: 1–40 years)		
≤ 9	101	39.0
–19	103	39.8
≥ 20	55	21.2
**Knowledge of HBV infection**		
Good	**163**	**62.9**
Inadequate	**96**	**37.1**
**Knowledge of HepB vaccine**		
Good	**170**	**65.6**
Inadequate	**89**	**34.4**
**Vaccination status**		
Received	**155**	**59.8**
Not Receive	**104**	**40.2**

*MD* Medical Doctor*, MA* Medical Assistant*, PHD* Provincial health department, *OD* Operational district, *PRH* Provincial referral hospital, *RH* Referral hospital, *HC* Health center, *OPD* Outpatient department, *ICU* Intensive care unit

Knowledge of HBV infection: inadequate knowledge < mean score value > good knowledge

Knowledge of HepB vaccine: inadequate knowledge < mean score value > good knowledge

Vaccination status (received): ≥ 1 dose

### Knowledge of HBV infection and associated factors

The mean score for knowledge of HBV infection was 7.3, with a standard deviation of 1.6. Among the 259 participants, the majority (62.9%) scored above the mean, indicating a good knowledge of HBV infection. Among participants, 61.4% of respondents were aware that HBV can be present in the semen or vaginal fluid of an infected person. Less than half (49%) accurately identified transmission routes, and 47.1% knew about preventive measures. More than half (57.9%) of respondents understood that HBV does not spread through the orofecal route or by eating; however, 42.1% were unaware of this fact. The vast majority (97.3%) knew that HBV infection could be prevented by receiving the vaccine. More than half of the participants understood that avoiding exposure to contaminated water (57.9%) and refraining from consuming uncooked food (53.3%) are not effective measures for preventing HBV transmission (Table 2).

**Table 2:** Knowledge about Hepatitis B infection among healthcare workers in Kampot and Kep, Cambodia

Knowledge items	Correct answer	
	**N**	**Percent (%)**
Hepatitis B infection can transmit through needle stick injury	244	94.2
Hepatitis B infection can be prevented by getting the vaccination	246	95.0
Hepatitis B virus can be found in the semen or vaginal fluid of the infected person	159	61.4
Hepatitis B–infected person can be asymptomatic	222	85.7
Every person exposed to the hepatitis B virus will develop acute hepatitis immediately	172	66.4
Hepatitis B virus is highly infectious	195	75.3
Only a small proportion of the world's population is infected with the hepatitis B virus	145	56.0
Hepatitis B virus mainly affects the liver	246	95.0
Hepatitis B virus can be transmitted from one person to the other through:	**127**	**49.0**
Sharps injury	240	92.7
Blood transfusion from the infected person	251	96.9
Sexual intercourse with the infected person	219	84.6
From mother to child during pregnancy/delivery	230	88.8
Oral-feces route	150	57.9
Contaminated water	213	82.2
Hepatitis B infection can be prevented by:	**122**	**47.1**
Vaccination	252	97.3
Using glove	247	95.4
Proper disposal of sharps	246	95.0
Avoiding multiple sexual partners	241	93.1
Avoiding expose to contaminated water	150	57.9
Avoiding uncooked food	138	53.3
**Overall**	**163**	**62.9**

The result of the multivariate analysis indicates that the knowledge of HBV infection among HCWs varies depending on occupation and knowledge of the HepB vaccine. Our data showed that physicians had 3.6 times higher knowledge of HBV infection compared to nurses (AOR: 3.6, 95% CI: 1.3–10.2, *p* = 0.0161). Additionally, HCWs with good knowledge of the HepB vaccine were 2.2 times more likely to possess knowledge of HBV infection compared to those with inadequate knowledge (AOR: 2.2, 95% CI: 1.2–3.8, *p* = 0.0063). Furthermore, our study did not find any statistically significant difference in knowledge of HBV infection across age, gender, provinces, workplace, working units, and work experience of HCWs (Table 3).

**Table 3:** Factors associated with knowledge of HBV infection among healthcare workers in Kampot and Kep, Cambodia

**Variables**	**Total** **N = 259**	**Knowledge**	**Univariate analysis**^**a**^			**Mutivariate analysis**^**b**^		
		**(Good)**						
		**n (%)**	**OR**	**[95%CI]**	***p*-value**	**AOR**	**[95%CI]**	***p*-value**
**Age (year)**								
≤ 30	57	33 (57.9)	0.6	[0.2–1.4]	0.2273	0.5	[0.2–1.2]	0.1236
31–40	132	83 (62.9)	0.7	[0.3–1.6]	0.4075	0.7	[0.3–1.7]	0.4827
41–50	33	21 (63.6)	0.7	[0.3–2.0]	0.5558	0.7	[0.2–1.9]	0.4753
> 50	37	26 (70.3)	1	(Reference)	–	1	(Reference)	–
**Gender**								
Female	144	84 (58.3)	1	(Reference)	–	–	–	–
Male	115	79 (68.7)	1.6	[0.9–2.6]	0.0871	–	–	–
**Occupation**								
Nurse	104	61 (58.7)	1	(Reference)	–	1	(Reference)	–
Midwife	99	57 (57.6)	0.9	[0.5–1.7]	0.8763	1.0	[0.6–1.8]	0.9506
Lab technician	9	6 (66.7)	1.4	[0.3–5.9]	0.6401	1.4	[0.3–6.1]	0.6482
Physician (MA/MD)	37	32 (86.5)	4.5	[1.6–12.5]	0.0038*	3.6	[1.3–10.2]	0.0161*
Others (Pharmacist & Administrator)	10	7 (70.0)	1.6	[0.4–6.7]	0.4884	2.2	[0.5–9.8]	0.2908
**Province**								
Kampot	232	148 (63.8)	1	(Reference)	–	–	–	–
Kep	27	15 (55.6)	0.7	[0.3–1.6]	0.4033	–	–	–
**Workplace**								
HCs	131	76 (58.0)	1	(Reference)	–	–	–	–
PRH/RHs	80	55 (68.7)	1.6	[0.9–2.9]	0.1201	–	–	–
PHD/ODs	48	32 (66.7)	1.4	[0.7–2.9]	0.2958	–	–	–
**Working unit**								
OPD/Other	98	60 (61.2)	1	(Reference)	–	–	–	–
Emergency/ICU/Surgery/Lab/Pediatric	40	29 (72.5)	1.7	[0.7–3.7]	0.2115	–	–	–
Obstetric/Delivery	82	48 (58.5)	0.9	[0.5–1.6]	0.7140	–	–	–
Technical/Admin/account	39	26 (66.7)	1.3	[0.6–2.8]	0.5525	–	–	–
**Experience (year)**								
≤ 9	101	65 (64.4)	0.9	[0.5–1.9]	0.8909	–	–	–
10–19	103	62 (60.2)	0.8	[0.4–1.6]	0.5167	–	–	–
≥ 20	55	36 (65.4)	1	(Reference)	–	–	–	–
**Knowledge of HepB vaccine**								
Good	170	119 (70.0)	2.4	[1.4–4.0]	0.0013	2.2	[1.2–3.8]	0.0063*
Inadequate	89	44 (49.4)	1	(Reference)	–	1	(Reference)	

Model *p* = 0.0057, r^2^ = 0.0632, N = 259, Age was always included in the model. The other seven variables were selected using the stepwise method (p < 0.25), – not selected by stepwise procedure

*MA* Medical assistant, *MD* Medical doctor, *PHD* Provincial health department, *OD* Operational district, *PRH* Provincial referral hospital, *RH* Referral hospital, *HC* Health center, *OPD* Outpatient department, *ICU* Intensive care unit, *HBV* Hepatitis B virus, *OR* Odds ratio, *AOR* Adjusted odds ratio, *CI* Confident interval

^*^Statistically significant

^a^Univariate analysis: χ^2^–test, ^b^Multivariate analysis: logistic regression

Knowledge of HepB vaccine: inadequate knowledge < mean score value > good knowledge

### Knowledge of HepB vaccine and associated factors

The mean score for knowledge of the HepB vaccine was 8.8, with a standard deviation of 2.1. Among the 259 participants, 65.6% scored above the mean, indicating good knowledge of the vaccine. Almost all participants (98.5%) were aware that the HepB vaccine is available for HBV prevention. More than half (59.1%) knew about its usefulness for post-exposure prophylaxis, but only 31.7% were aware of its effectiveness if administered within 24 h after exposure. Less than half (46.7%) knew that the vaccine could be given to immunocompromised patients, 40.2% knew that a blood test was not needed to confirm immunity after completing the full dose, and 41.3% were aware that the protection offered by a full dose of the vaccine lasting at least 20 years. Similarly, 41.3% knew that the vaccine could be administered to pregnant women. The majority (61.4%) were aware that the HepB vaccine is recommended for all HCWs (Table 4).

**Table 4:** Knowledge about Hepatitis B vaccine among healthcare workers in Kampot and Kep, Cambodia

Knowledge items	Correct answer	
	**N**	**Percent (%)**
An effective vaccine is available to prevent hepatitis B infection	255	98.5
The hepatitis B vaccine is useful for postexposure prophylaxis	153	59.1
Hepatitis B vaccine cannot be given to immune–compromised patients	121	46.7
The hepatitis B vaccine is effective for the treatment of acute hepatitis B infection	175	67.6
The hepatitis B vaccine can prevent hepatitis B infection effectively if given within 24 h after exposure	82	31.7
Healthcare workers should receive the hepatitis B vaccine as part of workplace safety	250	96.5
Full immunization in an adult consists of three or more doses of the hepatitis B vaccine	221	85.3
A full course of hepatitis B vaccine may give lifelong immunity, but a booster dose is recommended after five years for healthcare workers	194	74.9
After completing the full dose of hepatitis B vaccine, the blood test is not needed to confirm immunity against the hepatitis B virus	104	40.2
A complete course of the hepatitis B vaccine offers almost 100% protection against the hepatitis B virus	201	77.6
The protection offered by a full–dose hepatitis B vaccine lasts for at least 20 years	107	41.3
Hepatitis B vaccine cause problems when given to immune people	142	54.8
The hepatitis B vaccine can be administered to pregnant women	107	41.3
The hepatitis B vaccine is recommended for all healthcare workers	159	61.4
**Overall**	**170**	**65.6**

The findings from the multivariate model revealed variations in knowledge about the HepB vaccine among HCWs based on their age, occupations, workplaces, working units, and familiarity with HBV infection. HCWs aged 30 years or younger were found to have greater knowledge about the HepB vaccine compared to those aged over 50 years (AOR: 4.0, 95% CI: 1.4–11.2, *p* = 0.0092). Moreover, physicians were 5.9 times more likely to possess knowledge about the HepB vaccine compared to nurses (AOR: 5.9, 95% CI: 1.3–27.3, *p* = 0.0241). Conversely, individuals in other occupations were less likely to know about the HepB vaccine compared to nurses (AOR: 0.2, 95% CI: 0.1–0.8, *p* = 0.0237). The study also found that HCWs working at PRH/RHs were 2.5 times more likely to possess knowledge about the HepB vaccine compared to those at HCs (AOR: 2.5, 95% CI: 1.2–5.3, *p* = 0.0110). Additionally, HCWs at PHD/ODs were 3.4 times more likely to possess knowledge compared to those at HCs (AOR: 3.4, 95% CI: 1.4–8.1, *p* = 0.0056). Furthermore, HCWs working in emergency, intensive care unit (ICU), surgery, laboratory, or pediatric units had 2.6 times higher odds of possessing good knowledge about the HepB vaccine compared to those in outpatient departments (OPD) or other units (OR: 2.6, 95% CI: 1.0–6.5, *p* = 0.0390). Those with good knowledge of HBV infection were also more likely to possess knowledge about the HepB vaccine compared to those with inadequate knowledge (AOR: 2.2, 95% CI: 1.2–3.9, *p* = 0.0072). Interestingly, no statistically significant difference in knowledge about the HepB vaccine was found across genders, provinces, and work experience of HCWs (Table 5).

**Table 5:** HBV vaccination knowledge and its associated factors among healthcare workers in Kampot and Kep, Cambodia

Variables	Total	Knowledge	Univariate analysis^a^			Mutivariate analysis^b^		
	**N = 259**	**(Good)**						
		**n (%)**	**OR**	**[95%CI]**	***p*-value**	**AOR**	**[95%CI]**	***p*-value**
**Age (Year)**								
≤ 30	57	44 (77.2)	2.3	[0.9–5.7]	0.0692	4.0	[1.4–11.2]	0.0092*
31–40	132	83 (62.9)	1.1	[0.5–2.4]	0.7048	1.6	[0.7–3.7]	0.3001
41–50	33	21 (63.6)	1.2	[0.4–3.1]	0.7202	1.3	[0.4–4.0]	0.5934
> 50	37	22 (59.5)	1	(Reference)	–	1	(Reference)	–
**Gender**								
Female	144	90 (62.5)	1	(Reference)	–	–	–	–
Male	115	80 (69.6)	1.4	[0.8–2.3]	0.2349	–	–	–
**Occupation**								
Nurse	104	65 (62.5)	1	(Reference)	–	1	(Reference)	–
Midwife	99	58 (58.6)	0.8	[0.5–1.5]	0.5685	0.8	[0.4–1.5]	0.5630
Lab technician	9	7 (77.8)	2.1	[0.4–10.6]	0.3696	0.9	[0.1–4.9]	0.8828
Physician (MA/MD)	37	35 (94.6)	10.5	[2.4–46.1]	0.0018	5.9	[1.3–27.3]	0.0241*
Others (Pharmacist & Administrator)	10	5 (50.0)	0.6	[0.2–2.2]	0.4418	0.2	[0.1–0.8]	0.0237*
**Province**								
Kampot	232	153 (65.9)	1	(Reference)	–	–	–	–
Kep	27	17 (63.0)	0.9	[0.4–2.0]	0.7573	–	–	–
**Workplace**								
HCs	131	70 (53.4)	1	(Reference)	–	1	(Reference)	–
PRH/RHs	80	63 (78.7)	3.2	[1.7–6.1]	0.0003	2.5	[1.2–5.3]	0.0110*
PHD/ODs	48	37 (77.1)	2.9	[1.4–6.2]	0.0053	3.4	[1.4–8.1]	0.0056*
**Working unit**								
OPD/Other	98	63 (64.3)	1	(Reference)	–	–	–	–
Emergency/ICU/Surgery/Lab/Pediatric	40	33 (82.5)	2.6	[1.0–6.5]	0.0390*	–	–	–
Obstetric/Delivery	82	45 (54.9)	0.7	[0.4–1.2]	0.2003	–	–	–
Technical/Admin/account	39	29 (74.4)	1.6	[0.7–3.7]	0.2595	–	–	–
**Experience**								
≤ 9	101	73 (72.3)	1	(Reference)	–	–	–	–
10–19	103	62 (60.2)	0.6	[0.3–1.0]	0.0693	–	–	–
≥ 20	55	35 (63.6)	0.7	[0.3–1.3]	0.2652	–	–	–
**Knowledge of HBV infection**								
Good	163	119 (73.0)	2.4	[1.4–4.0]	0.0013	2.2	[1.2–3.9]	0.0072*
Inadequate	96	51 (53.1)	1	(Reference)	–	1	(Reference)	–

Note: Model p < .0001, r^2^ = 0.1429, N = 259, Age was always included in the model. The other seven variables were selected using the stepwise method (p < 0.25), – not selected by stepwise procedure

*MA* Medical assistant, *MD* Medical doctor, *PHD* Provincial health department, *OD* Operational district, *PRH* Provincial referral hospital, *RH* Referral hospital, *HC* Health center, *OPD* Outpatient department, *ICU* Intensive care unit, *HBV* Hepatitis B virus, *OR* Odds ratio, *AOR* Adjusted odds ratio, *CI* Confident interval

^*^Statistically significant

^a^Univariate analysis: χ^2^–test, ^b^Multivariate analysis: logistic regression

Knowledge of HBV infection: inadequate knowledge < mean score value > good knowledge

### HepB vaccination status and associated factors

**Fig. 2:**
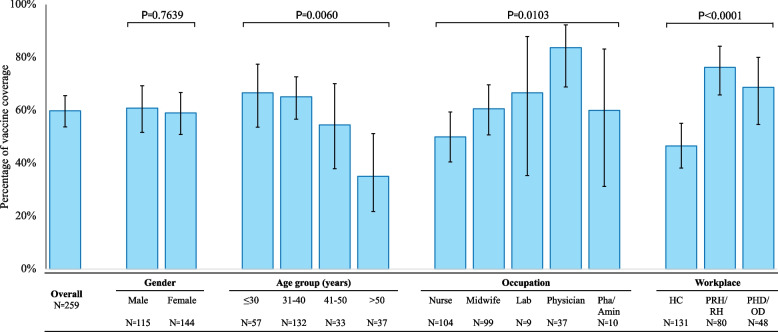
Hepatitis B Vaccination Coverage. This bar graph delineates the hepatitis B vaccination coverage rates among healthcare workers, stratified by gender, age group, occupation, and workplace. The horizontal axis categorizes the data into total coverage proportion, gender (Male, Female), age groups (years), occupations (Nurse, Midwife, Lab Technician, Physician, Pharmacist, Administrator), and workplaces (Health Center, Provincial Referral Hospital/Referral Hospital, Provincial Health Department/Operational District). The vertical axis quantifies the percentage of vaccination coverage. The graph elucidates variations in coverage rates across the demographics, underscored by statistical evaluations with p-values. The sample sizes for each subgroup are explicitly stated (N), and error bars depict the variability within each demographic segment

Among the 259 individuals surveyed, 59.8% have received at least one dose of the HepB vaccine. Of those vaccinated, 82.6% were fully vaccinated. The vaccination coverage rates are comparable between males (60.9%) and females (59%). However, the coverage rate is highest among HCWs aged 30 or under, at 66.7%, and lowest among those aged 50, at 35.1%. Notably, physicians exhibit the highest coverage rate at 83.8%, while nurses show a lower coverage rate of 50%. The coverage rates at HCs, PRH/RHs, and PHD/ODs were 46.6%, 76.2%, and 68.7%, respectively (Fig. 2).

The multivariate model showed that vaccination coverage among HCWs is influenced by various factors such as age, workplace, years of experience, knowledge of HBV infection, and familiarity with the HepB vaccine. HCWs aged 30 years or younger are 7.9 times more likely to be vaccinated compared to those aged over 50 years (AOR: 7.9, 95% CI: 2.5–25.4, *p* = 0.0005). Similarly, HCWs aged 31–40 years and 41–50 years are more likely to be vaccinated compared to those aged over 50 years (AOR: 7.4, 95% CI: 2.7–20.1, *p* < 0.0001; AOR: 3.8, 95% CI: 1.1–12.5, *p* = 0.0304), respectively. The odds of HCWs working at PRH/RHs receiving the HepB vaccine are 2.8 times greater than those at HCs (AOR: 2.8, 95% CI: 1.0–7.8, *p* = 0.0412), while the odds of those working at PHD/ODs receiving the HepB vaccine are 6.5 times greater compared to those at HCs (AOR: 6.5, 95% CI: 1.1–39.5, *p* = 0.0403). HCWs with 9 years or less of work experience are more likely to receive the vaccine compared to those with 20 years or longer of experience (OR: 2.9, 95% CI: 1.5–5.7, *p* = 0.0020). Those with good knowledge of HBV infection are 6.3 times more likely to be vaccinated compared to those with inadequate knowledge (AOR: 6.3, 95% CI: 3.3–12.3, *p* < 0.0001). Similarly, those with good knowledge of the HepB vaccine are 3.7 times more likely to receive the vaccine compared to those with inadequate knowledge (AOR: 3.7, 95% CI: 1.9–7.4, *p* = 0.0001). In the crude analysis, physicians are 5.2 times more likely to be vaccinated compared to nurses (OR: 5.2, 95% CI: 2.0–13.4, *p* = 0.0008). Additionally, those working in emergency, ICU, surgery, lab, or pediatric units are 3.6 times more likely to be vaccinated compared to those at OPD or other units (OR: 3.6, 95% CI: 1.5–8.3, *p* = 0.0029). However, after adjusting for odds ratio, there was no statistically significant difference (*p* > 0.05). Furthermore, this study found no statistically significant difference in vaccine uptake concerning gender and the provinces where HCWs have worked, as shown in (Table 6).

**Table 6:** HBV vaccination status and its associated factors among healthcare workers in Kampot and Kep, Cambodia

Variables	Total **N = 259**	Vaccine	Univariate analysis^a^			Mutivariate analysis^b^		
		**(Receive)**						
		**n (%)**	**OR**	**[95%CI]**	***p*-value**	**AOR**	**[95%CI]**	***p*-value**
**Age (year)**								
≤ 30	57	38 (66.7)	3.7	[1.5–8.8]	0.0033	7.9	[2.5–25.4]	0.0005*
31–40	132	86 (65.1)	3.4	[1.6–7.4]	0.0015	7.4	[2.7–20.1]	< .0001*
41–50	33	18 (54.5)	2.2	[0.8–5.8]	0.1050	3.8	[1.1–12.5]	0.0304*
> 50	37	13 (35.1)	1	(Reference)	–	1	(Reference)	–
**Gender**								
Female	144	85 (59.0)	1	(Reference)	–	–	–	–
Male	115	70 (60.9)	1.1	[0.6–1.8]	0.7639	–	–	–
**Occupation**								
Nurse	104	52 (50.0)	1	(Reference)	–	1	(Reference)	–
Midwife	99	60 (60.6)	1.5	[0.9–2.7]	0.1296	0.9	[0.3–3.1]	0.9086
Lab technician	9	6 (66.7)	2.0	[0.5–8.4]	0.3449	0.5	[0.1–2.7]	0.4127
Physician (MA/MD)	37	31 (83.8)	5.2	[2.0–13.4]	0.0008	2.0	[0.6–6.2]	0.2587
Others (Pharmacist & Administrator)	10	6 (60.0)	1.5	[0.4–5.6]	0.5478	0.7	[0.1–4.1]	0.6651
**Province**								
Kampot	232	138 (59.5)	1	(Reference)	–	–	–	–
Kep	27	17 (63.0)	1.1	[0.5–2.6]	0.7272	–	–	–
**Workplace**								
HCs	131	61 (46.6)	1	(Reference)	–	1	(Reference)	–
PRH/RHs	80	61 (76.2)	3.7	[2.0–6.8]	< .0001	2.8	[1.0–7.8]	0.0412*
PHD/ODs	48	33 (68.7)	2.5	[1.2–5.1]	0.0095	6.5	[1.1–39.5]	0.0403*
**Working unit**								
OPD/Other	98	48 (49.0)	1	(Reference)	–	1	(Reference)	–
Emergency/ICU/Surgery/Lab/Pediatric	40	31 (77.5)	3.6	[1.5–8.3]	0.0029	1.3	[0.3–4.8]	0.7110
Obstetric/Delivery	82	50 (61.0)	1.6	[0.9–2.9]	0.1084	2.6	[0.7–9.7]	0.1547
Technical/Admin/account	39	26 (66.7)	2.1	[1.0–4.5]	0.0633	0.6	[0.1–3.7]	0.6133
**Experience (year)**								
≤ 9	101	70 (69.3)	2.9	[1.5–5.7]	0.0020*	–	–	–
10–19	103	61 (59.2)	1.9	[1.0–3.6]	0.0626	–	–	–
≥ 20	55	24 (43.6)	1	(Reference)	–	–	–	–
**Knowledge of HBV infection**								
Good	163	121 (74.2)	5.2	[3.0–9.1]	< .0001	6.3	[3.3–12.3]	< .0001*
Inadequate	96	34 (35.4)	1	(Reference)	–	1	(Reference)	–
**Knowledge of HepB vaccine**								
Good	170	124 (72.9)	5.0	[2.9–8.7]	< .0001	3.7	[1.9–7.4]	0.0001*
Inadequate	89	31 (34.8)	1	(Reference)	–	1	(Reference)	–

Note: Model *p* < .0001, r^2^ = 0.2872, N = 259, Age was always included in the model. The other eight variables were selected using the stepwise method (p < 0.25), – not selected by stepwise procedure, * Statistically significant

*MA* Medical assistant, *MD* Medical doctor, *PHD* Provincial health department, *OD* Operational district, *PRH* Provincial referral hospital, *RH* Referral hospital, *HC* Health center, *OPD* Outpatient department, *ICU* Intensive care unit, *HBV* Hepatitis B virus, *OR* Odds ratio, *AOR* Adjusted odds ratio, *CI* Confident interval

^a^Univariate analysis: χ^2^–test, ^b^Multivariate analysis: logistic regression. Knowledge of HBV infection: inadequate knowledge < mean score value > good knowledge. Knowledge of HepB vaccine: inadequate knowledge < mean score value > good knowledge

### Reasons for being unvaccinated

Among the HCWs surveyed, 40.2% did not receive HepB vaccine. Of the unvaccinated individuals, 5.8% were unaware of adult HepB vaccination, 32.7% mentioned that the vaccine at their workplaces is not provided for adults, 31.7% reported high vaccine costs, 5.8% lacked time for vaccination, 1.9% tested positive for HBV infection, 18.3% already had anti-HBs antibodies, and 58.6% indicated insufficient education on adult HepB vaccination (Supplementary Table 1).

### History of occupational exposure

During the last 12 months, 149 (57.5%) HCWs reported being exposed to blood or body fluids on intact skin and 35.6% of them had not received the HepB vaccine. Additionally, 40 (15.4%) HCWs reported incidents of blood or body fluids splashing into their eyes or mouth, with 25% of them unvaccinated. Moreover, 51 (19.7%) HCWs experienced accidental exposure to blood or bodily fluids on a cut or scratched skin surface, and 33.3% of them were not vaccinated against HBV (Supplementary Table 2).

## Discussion

Our study revealed that 62.9% of HCWs demonstrated good knowledge of HBV infection, which is below the expectation that all HCWs should have such knowledge [6, 26]. However, our finding is slightly higher compared to a study using similar tools conducted in Northwest Ethiopia where 52% demonstrated good knowledge [6]​. Furthermore, almost all participants were aware that HBV infection can be prevented by vaccination, consistent with a nationwide study in 2019 [13]. Nonetheless, nearly half mistakenly believed that HBV could spread through the orofecal route, potentially leading to stigma and discrimination against infected individuals [27]. Additionally, less than half of the participants were knowledgeable about transmission routes and prevention measures, posing a risk of HBV infection among HCWs. There is an urgent need for education on HBV transmission and prevention for HCWs [23]. It highlights the necessity of viral hepatitis education programs to enhance awareness among HCWs. This can be achieved by integrating the content of viral hepatitis prevention and treatment into the existing training curriculum, providing in-service training, and incorporating it into the National Infection Prevention and Control (IPC) program as introduced in Cambodia’s first national strategic plan (2020–2024) for viral hepatitis B and C infection control [13, 28].

In our study, physicians showed significantly greater knowledge of HBV infection compared to nurses, possibly due to physicians' education curriculum covering in-depth information about various infections, including hepatitis. This finding is consistent with a study conducted in Sierra Leone [23]. There was no statistically significant difference in knowledge of HBV infection across age, gender, provinces, workplaces, working units, and work experience of HCWs, in line with the report in Northern Vietnam [27]. The HBV training program should be provided to all HCWs, with particular emphasis on nurses, midwives, and laboratory technicians.

Our study revealed that 65.6% of HCWs exhibited good knowledge of the HepB vaccine. This observation aligns with the reported rate in Northwest Ethiopia (62%) using similar assessment tools [6]​​.​ Of all participants, 59.1% were aware of the vaccine’s utility for post-exposure prophylaxis. This lack of awareness on this issue is concerning as it may lead to missed vaccination opportunities for unvaccinated individuals following exposure [12]. Notably, a quarter of those who reported blood or body fluids splashing into their eyes or mouths were not vaccinated. Additionally, one-third of those who experienced accidental exposure to blood or bodily fluids on cut or scratched skin were also unvaccinated against HBV. These individuals could be immunized for post-exposure prophylaxis (PEP) [29]. Immunocompromised individuals are particularly vulnerable to HBV infection and are at a higher risk of rapid progression to liver cirrhosis and hepatocellular carcinoma (HCC). Vaccination against HBV is strongly recommended for this population [30, 31]. However, less than half of the participants were aware that the vaccine could be administered to immunocompromised patients. This lack of awareness may leave these patients unprotected. Our findings also revealed that 41.3% of the participants knew that a full dose of the vaccine provides protection for at least 20 years. Although this represents a slight improvement compared to a prior report (37.1%) [13], further enhancements are necessary. Only two-fifths were aware that the HepB vaccine can be administered to pregnant women. This limited awareness may lead to missed vaccination opportunities for unvaccinated pregnant individuals. This finding highlights a significant knowledge gap on HepB immunization. These findings underscore the importance of educational programs on HepB vaccination to enhance knowledge among HCWs.

The study indicated that physicians have a greater knowledge of the HepB vaccine compared to nurses, potentially due to the more extensive education physicians receive on infectious diseases, treatments, and vaccines compared to nurses, who primarily focus on patient care. Those with good knowledge of infections also tend to have better understanding of the vaccine, suggesting that individuals with adequate knowledge may be more informed about HBV prevention, vaccination, and its benefits when compared to those with insufficient knowledge. Education could give priority to nurses and other HCWs rather than physicians.

Our present study revealed that 59.8% of HCWs were vaccinated against HBV, showing an increase from the 2019 nationwide study which reported 46.7% [13]. This percentage is slightly lower than the neighboring Southeast Asian countries such as Thailand (68%) [32] and Vietnam (83%) [19], but higher than Lao PDR (52.6%) [33] and other African countries, including Ethiopia (10%) [6]​, Cameroon (13.9%) ​[26], Nigeria (46.7%) [12], and Tanzania (56.9%) ​[34]​. However, this rate is below those observed in developed countries like Japan (84%) [35] and China (86%) [36]. Our reported rate does not meet the recommendations of the World Health Organization (WHO) and the Centers for Disease Control and Prevention (CDC), which advocate that all HCWs should receive vaccine against HBV infection [29, 30]. Improving the vaccination status among HCWs could significantly contribute to reducing healthcare-associated transmission of viral hepatitis [28].

In our study, we observed that vaccination rates decreased with age, aligning with the previous reports in Cambodia [13] and China [36]. Gender did not show any significant variation in vaccination rates, consistent with reports from China [36] and Cameroon [26]. Our study revealed that physicians had a higher vaccination rate (83.8%) compared to nurses (50%), which is in line with a Vietnamese study ​[37]​. The crude analysis suggested that physicians were 5.2 times more likely to be vaccinated than nurses (*p* = 0.0008), in line with previous findings [13]. However, after adjusting the odds ratio, no significant difference was observed (*p* = 0.2587), possibly due to sample size limitations. These results underscore the need for larger studies to provide more robust insights into vaccination patterns. Those working at higher-level HFs had significantly higher vaccination rates compared to those at primary HFs, which can be attributed to greater HepB vaccine knowledge among them. Interestingly, our study found that HCWs with better knowledge of HBV infection or vaccination are more likely to be vaccinated. These findings are consistent with the recent studies conducted in Cameroon and China [26, 36]. Notably, our participants were at least 24 years old at the time of the study and were born before the introduction of the newborn HepB vaccination program in 2001, which was later implemented nationwide in 2005 [4], indicating that they did not receive this vaccine at that time. Of the unvaccinated HCWs, approximately 83% intended to be vaccinated. However, one-third cited high vaccination costs, another one-third mentioned that the vaccine available at their workplace is not for adults, and half cited insufficient education on adult HepB vaccination as reasons for not being vaccinated. High vaccine costs have been previously reported as a barrier for public HCWs in Cambodia [13]. This issue has also been noted in other countries such as Vietnam and China [36, 37]. Therefore, there is a need for a national policy providing free vaccination for HCWs [13]. Alongside education on HBV infection and vaccination, this policy could potentially improve vaccination rates and reduce the risk of infection among HCWs.

This study has several limitations. Firstly, the use of self-reporting for HepB vaccination may introduce recall bias, leading to a potential overestimation or underestimation of the reported vaccination rate. Secondly, knowledge questionnaires may focus on theoretical understanding without fully assessing practical knowledge or the ability to apply it in real-world situations, which is crucial for informed decision-making on prevention. Lastly, the study's scope was restricted to just two out of twenty-five provinces and municipalities. Despite these limitations, the study furnishes valuable insights into HepB vaccination coverage among HCWs. The use of systematic random sampling ensured the participation of HCWs from all 83 HFs, these participants are well representative of all HCWs in Kampot and Kep**.**

## Conclusions

The study revealed that HepB vaccination coverage among HCWs is 59.8%, which is slightly lower than in neighboring countries and below the World Health Organization's (WHO) recommendation. The knowledge of HBV infection and the HepB vaccine were significant predictors of vaccination. HCWs with good knowledge of HBV infection are 6.3 times more likely to be vaccinated, and those with good knowledge of the HepB vaccine are 3.7 times more likely to be vaccinated compared to those with inadequate knowledge. The main barriers to vaccination among HCWs include high vaccine cost, workplace vaccines not provided for adults, and insufficient education on adult vaccination. The study suggests the need for education on HBV infection and the HepB vaccine. Furthermore, the study emphasizes the need to implement a national policy for free HepB vaccination for HCWs.

### Supplementary Information

Supplementary Material 1.

Supplementary Material 2.

## Data Availability

The datasets used and analyzed during the current study are available from the corresponding author upon reasonable request.

## Abbreviations

CHB: Chronic Hepatitis B
HBsAb: Hepatitis B Surface Antibody
HBV: Hepatitis B Virus
HCs: Health Centers
HCWs: Healthcare Workers
HepB: Hepatitis B
HFs: Health Facilities
MoH: Ministry of Health
NECHR: National Ethics Committee for Health Research
ODs: Operational Districts
PHDs: Provincial Health Departments
PRHs: Provincial Referral Hospitals
RHs: Referral Hospitals
WHO: World Health Organization
