# Supplementary material for: Knowledge of hepatitis B infection, hepatitis B vaccine, and vaccination status with its associated factors among healthcare workers in Kampot and Kep Provinces, Cambodia
Source: BMC Infect Dis. 2024 Jul 1;24:658. doi: 10.1186/s12879-024-09571-y (PMC11218137; doi:10.1186/s12879-024-09571-y)
Supplement: Supplementary file 1 — Supplementary Material 1. [file 12879_2024_9571_MOESM1_ESM.docx]

**Annex1** Scoring Checklist

| **Knowledge about Hepatitis B infection among healthcare workers in Kampot and Kep, Cambodia** | | |
| --- | --- | --- |
| **Knowledge items** | **Response** | **Correct answer** |
| Hepatitis B infection can transmit through needle stick injury | Yes No Don’t know | Yes |
| Hepatitis B infection can be prevented by getting the vaccination | Yes No Don’t know | Yes |
| Hepatitis B virus can be found in the semen or vaginal fluid of the infected person | Yes No Don’t know | Yes |
| Hepatitis B infected person can be asymptomatic | Yes No Don’t know | Yes |
| Every person exposed to the hepatitis B virus will develop acute hepatitis immediately | Yes No Don’t know | No |
| Hepatitis B virus is highly infectious | Yes No Don’t know | Yes |
| Only a small proportion of the world's population is infected with the hepatitis B virus | Yes No Don’t know | Yes |
| Hepatitis B virus mainly affects the liver | Yes No Don’t know | Yes |
| Hepatitis B virus can be transmitted from one person to the other through:  Sharps injury  Blood transfusion from the infected person  Sexual intercourse with the infected person  From mother to child during pregnancy/delivery  Oral-feces route  Contaminated water | Yes No Don’t know  Yes No Don’t know  Yes No Don’t know  Yes No Don’t know  Yes No Don’t know  Yes No Don’t know | Yes  Yes  Yes  Yes  No  No |
| Hepatitis B infection can be prevented by:  Vaccination  Using glove  Proper disposal of sharps  Avoiding multiple sexual partners  Avoiding exposure to contaminated water  Avoiding uncooked food | Yes No Don’t know  Yes No Don’t know  Yes No Don’t know  Yes No Don’t know  Yes No Don’t know  Yes No Don’t know | Yes  Yes  Yes  Yes  No  No |

*Remark: One correct answer was given 1 point, while an incorrect answer or "don't know" was given zero. For questions 9 and 10, if answered correctly all points were given 1.*

| **Knowledge about Hepatitis B vaccine among healthcare workers in Kampot and Kep, Cambodia** | | |
| --- | --- | --- |
| **Knowledge items** | **Response** | **Correct answer** |
| An effective vaccine is available to prevent hepatitis B infection | Yes No Don’t know | Yes |
| The hepatitis B vaccine is useful for postexposure prophylaxis | Yes No Don’t know | Yes |
| Hepatitis B vaccine cannot be given to immune–compromised patients | Yes No Don’t know | No |
| The hepatitis B vaccine is effective for the treatment of acute hepatitis B infection | Yes No Don’t know | No |
| The hepatitis B vaccine can prevent hepatitis B infection effectively if given within 24h after exposure | Yes No Don’t know | Yes |
| Healthcare workers should receive the hepatitis B vaccine as part of workplace safety | Yes No Don’t know | Yes |
| Full immunization in an adult consists of three or more doses of the hepatitis B vaccine | Yes No Don’t know | Yes |
| A full course of hepatitis B vaccine may give lifelong immunity, but a booster dose is recommended after five years for healthcare workers | Yes No Don’t know | Yes |
| After completing the full dose of the hepatitis B vaccine, the blood test is not needed to confirm immunity against the hepatitis B virus | Yes No Don’t know | Yes |
| A complete course of the hepatitis B vaccine offers almost 100% protection against the hepatitis B virus | Yes No Don’t know | Yes |
| The protection offered by a full–dose hepatitis B vaccine lasts for at least 20 years | Yes No Don’t know | Yes |
| Hepatitis B vaccine cause problems when given to immune people | Yes No Don’t know | No |
| The hepatitis B vaccine can be administered to pregnant women | Yes No Don’t know | Yes |
| The hepatitis B vaccine is recommended for all healthcare workers | Yes No Don’t know | Yes |

*Remark: One correct answer was given 1 point, while an incorrect answer or don't know was given zero.*
