# Supplementary material for: Knowledge of hepatitis B infection, hepatitis B vaccine, and vaccination status with its associated factors among healthcare workers in Kampot and Kep Provinces, Cambodia
Source: BMC Infect Dis. 2024 Jul 1;24:658. doi: 10.1186/s12879-024-09571-y (PMC11218137; doi:10.1186/s12879-024-09571-y)
Supplement: Supplementary file 2 — Supplementary Material 2. [file 12879_2024_9571_MOESM2_ESM.docx]

| **Supplementary Table 1. Reasons for being unvaccinated among healthcare workers in Kampot and Kep, Cambodia** | | |
| --- | --- | --- |
| **Description** | **Response** | |
|  | **Yes (%)** | **No (%)** |
| Have you received the hepatitis B vaccine before? (N=259) | 155 (59.8) | 104 (40.2) |
| If yes, how many doses did you receive? (n=155)  1 dose  2 doses  ≥ 3 doses | 10 (6.5)  17 (11.0)  128 (82.6) | –  –  – |
| If no, do you intend to receive it in the future? (n=104) | 86 (82.7) | 18 (17.3) |
| If never been vaccinated, please specify (n=104)  – I never heard about the vaccination before  – The vaccine at my workplace is not available for adults  – High cost of the vaccine  – Lack of time  – I was found with hepatitis B infection on initial screening  – I already had anti-HBs on screening  – There is not enough education regarding hepatitis B vaccination for adult | 6 (5.8)  34 (32.7)  33 (31.7)  6 (5.8)  2 (1.9)  19 (18.3)  61 (58.6) | –  –  –  –  –  –  – |

| **Supplementary Table 2. History of occupational exposure during the last 12 months among healthcare workers in Kampot and Kep, Cambodia** | | | |
| --- | --- | --- | --- |
| **Description** | **Vaccinated**  **n (%)** | **Unvaccinated**  **n (%)** | **Total**  **N=259**  **n (%)** |
| Exposure to blood or body fluids on intact skin (n=149) | 96 (64.4) | 53 (35.6) | 149 (57.5) |
| Splash of blood or body fluids to the eye or mouth (n=40) | 30 (75.0) | 10 (25.0) | 40 (15.4) |
| Exposure of blood or body fluids on cut or scratched skin (n=51) | 34 (66.7) | 17 (33.3) | 51 (19.7) |
